# (p)ppGpp – an important player during heat shock response

**DOI:** 10.1093/femsml/uqad017

**Published:** 2023-04-06

**Authors:** Kristina Driller, Fabián A Cornejo, Kürşad Turgay

**Affiliations:** Max Planck Unit for the Science of Pathogens, Charitéplatz 1, 10117 Berlin, Germany; Leibniz Universität Hannover, Institut für Mikrobiologie, Herrenhäuser Str. 2, 30419 Hannover, Germany; Max Planck Unit for the Science of Pathogens, Charitéplatz 1, 10117 Berlin, Germany; Max Planck Unit for the Science of Pathogens, Charitéplatz 1, 10117 Berlin, Germany; Leibniz Universität Hannover, Institut für Mikrobiologie, Herrenhäuser Str. 2, 30419 Hannover, Germany

**Keywords:** stringent response, heat shock response, *Bacillus subtilis*, second messenger, Rel (RSH), (p)ppGpp

## Abstract

The alarmones and second messengers (p)ppGpp are important for the cellular response to amino acid starvation. Although the stringent response is present in many bacteria, the targets and functions of (p)ppGpp can differ between species, and our knowledge of (p)ppGpp targets is constantly expanding. Recently, it was demonstrated that these alarmones are also part of the heat shock response in *Bacillus subtilis* and that there is a functional overlap with the oxidative and heat stress transcriptional regulator Spx. Here, the (p)ppGpp second messenger alarmones allow the fast stress-induced downregulation of translation while Spx inhibits the further expression of translation-related genes to lower the load on the protein quality control system, while the chaperone and protease expression is induced. In this review, we discuss the role of (p)ppGpp and its intricate connections in the complex network of stress sensing, heat shock response, and adaptation in *B. subtilis* cells.

## Teaser

In this review we discuss the interplay of stringent and heat stress response in *Bacillus subtilis*.

## Introduction

Adaptation of bacteria to their environment is crucial for their survival. Many environmental changes, such as osmolarity, temperature, and pH fluctuations, can often lead to misfolded proteins and protein aggregation. Therefore, the evolutionarily conserved protein quality control (PQC) system is essential for all living organisms to maintain protein homeostasis and prevent the accumulation of toxic protein aggregates. PQC systems are part of the cellular heat shock response, a regulatory and signal transduction network that allows response and adaptation to such proteotoxic stresses. This response varies between the model organisms for Gram-negative and Gram-positive bacteria, *Escherichia coli*, and *Bacillus subtilis*. In *E. coli*, the expression of cytosolic chaperones and proteases is transcriptionally activated by the heat shock sigma factors H. In contrast, the expression of chaperones and proteases in *B. subtilis* is regulated by the transcriptional repressors CtsR and HrcA, the transcriptional regulator Spx and the general stress sigma factor B (SigB), which are discussed later in this review (Hecker et al. 1996, Lim and Gross 2010, Elsholz et al. 2017, Schramm et al. 2020, Matavacas and von Wachenfeldt 2022).

The alarmones guanosine pentaphosphate and guanosine tetraphosphate, collectively called (p)ppGpp, were identified as crucial second messengers during amino acid starvation, but they are also important during many other stresses. These second messenger molecules are conserved throughout most bacterial species to mitochondria and chloroplasts (Atkinson et al. 2011, Ito et al. 2020). However, the targets and functions of (p)ppGpp, the regulation and interaction partners of the respective synthetase or hydrolase enzyme domains, can differ between species and are adapted to their specific ecological niche and environments (Boutte and Crosson 2013, Liu et al. 2015, Bange et al. 2021, Irving et al. 2021). When comparing the model organisms *E. coli* and *B. subtilis*, it can be observed that the targets of the translation inhibition are similar; however, the transcriptional regulation is controlled by different mechanisms (Travis and Schumacher 2022). In this review, we discuss protein folding stresses, such as raised temperatures and how the alarmones (p)ppGpp integrate into these stress responses in *B. subtilis*.

### Proteotoxic stress response regulation in *B. subtilis*

One of the main problems when facing heat, salt, or oxidative stress is the maintenance of correct protein folding. In *B. subtilis*, the different classes of heat shock proteins are transcriptionally regulated by the general stress sigma factor SigB, the transcriptional regulator Spx and the two more specific repressors, HrcA and CtsR. (Hecker et al. 1996, Elsholz et al. 2017).

#### HrcA and protein repair

**Figure 1.**
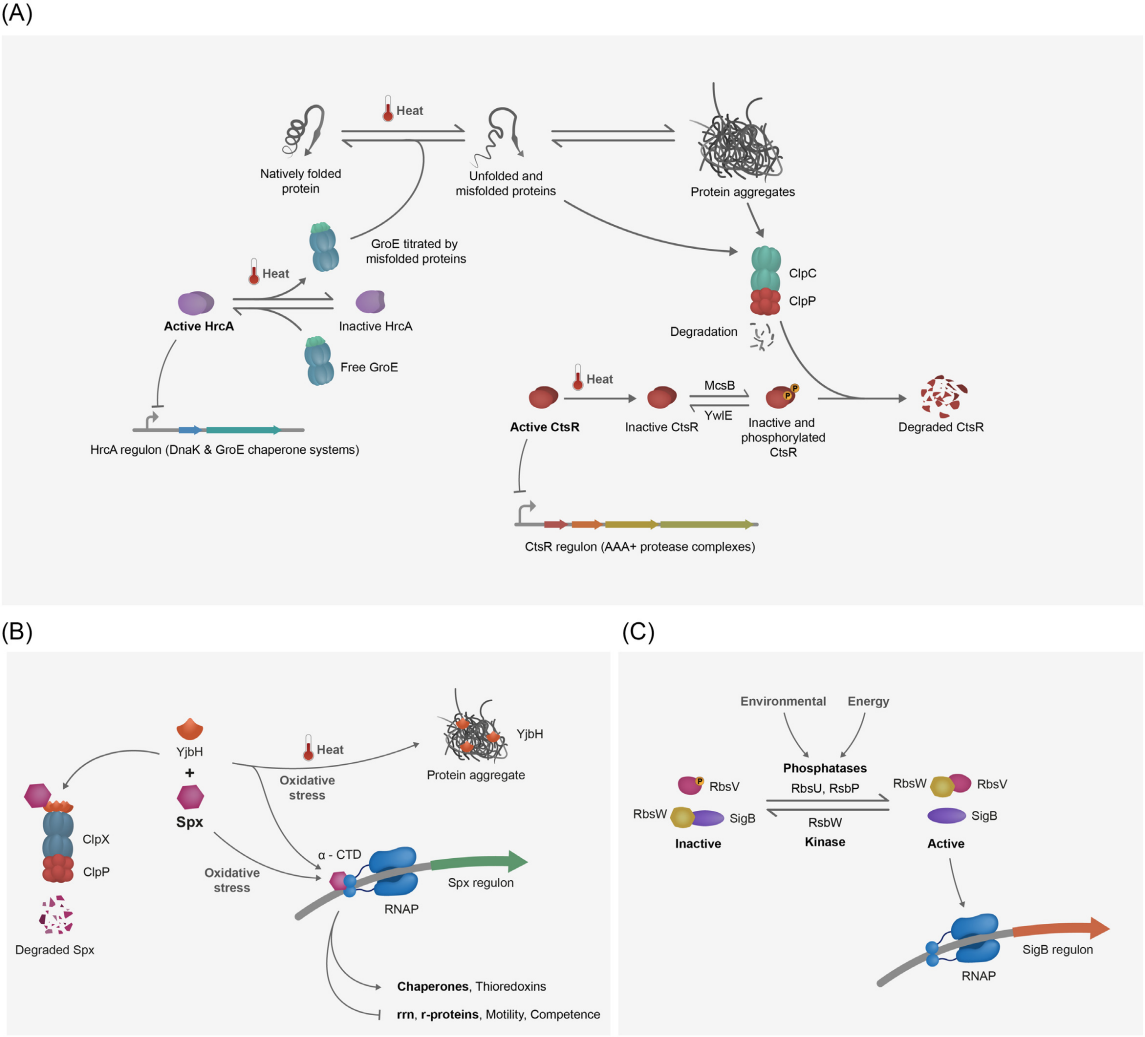
Regulation of transcription in *B. subtilis* upon heat shock. Heat shock and other proteotoxic stresses lead to protein misfolding and aggregation. **(A)** The transcriptional repressor HrcA is kept in its active conformation by the chaperonin GroE, repressing the transcription of its target promoters. GroE is titrated by misfolded proteins, inactivating HrcA and allowing chaperone transcription. The transcriptional repressor CtsR controls the expression of AAA+ protease complexes. CtsR is primarily inactivated by heat, phosphorylated on arginine residues by McsB, and targeted for proteolysis by ClpCP. **(B)** During nonstress conditions, the adaptor protein YjbH targets Spx for degradation by ClpXP. Upon protein folding stress, YjbH localizes with protein aggregates, resulting in Spx stabilization. Spx interacts with the α-CTD subunit of the RNA polymerase to modulate transcription of its regulon. The cellular redox state can also tune Spx activity. **(C)** SigB is a general stress sigma factor for the RNA polymerase. During nonstress conditions, SigB is sequestered by the antisigma factor RsbW, impeding its interaction with the RNA polymerase. When environmental or energy stress is sensed, the phosphatases RbsU or RbsP are activated and dephosphorylate the anti-anti-sigma factor RbsV, which binds RsbW, releasing SigB, activating the transcription of the SigB regulon.

HrcA regulates the expression of chaperone proteins (Fig. 1A; left-hand side). Under nonstress conditions, HrcA binds to promoters containing the CIRCE (Controlling Inverted Repeat of Chaperone Expression) elements, repressing the transcription of two operons, the *dnaK* operon that also encodes the *hrcA* and the *groES–groEL* operon. HrcA is translated in an inactive conformation unable to bind to its target promoters. During nonstress conditions, free GroE chaperonin (consisting of GroEL and GroES) stabilizes the active conformation of HrcA (Zuber and Schumann 1994, Mogk 1997, Reischl et al. 2002). Upon proteotoxic stress, the GroE chaperonin switches to interact with the increased unfolded proteins and, therefore, cannot maintain the repressor activity of HrcA anymore. This results in the expression of the chaperone systems DnaKJE and GroE (Mogk 1997). These two chaperone systems prevent protein aggregation of misfolded or unfolded substrate proteins, which they can recognize by exposed patches of hydrophobic amino acids usually inside globular proteins. Both chaperone systems can facilitate the refolding of their substrate proteins by distinct mechanisms driven by an ATPase cycle (Bukau and Horwich 1998). Upon clearance of unfolded misfolded proteins, the repression of chaperones by HrcA is restored by free GroE (Mogk 1997).

#### CtsR and degradation of misfolded and aggregated proteins

CtsR (Class three stress repressor) regulates another class of heat shock proteins, the AAA+ protease complexes (Fig. 1A; right-hand side). Like HrcA, CtsR represses the transcription of its targets by binding to their promoter region in the absence of stress. In *B. subtilis*, CtsR has an intrinsic thermosensitive tetra-glycine loop, which changes its conformation upon temperature upshift (Fuhrmann et al. 2009, Elsholz et al. 2010), resulting in de-repression of the CtsR regulon consisting of the *clpC* operon (*ctsR*–*mcsA*–*mcsB*–*clpC*) and the genes for *clpP* and *clpE*. CtsR, thereby controls the expression of the ClpCP and ClpEP AAA+ protease complex and the McsB and McsA adaptor protein complex for ClpC. Interestingly, the inactive CtsR is degraded, but the newly synthesized CtsR can rebind the operator element when the stress conditions cease, returning the CtsR regulon to the repressed state (Krüger and Hecker 1998, Derre et al. 1999b). The AAA+ protease complexes ClpCP and ClpEP are critical for survival during heat shock response as they can recognize and degrade misfolded/aggregated proteins (Krüger et al. 2000, Gerth et al. 2004, Miethke et al. 2006). ClpCP-dependent protein degradation can be regulated by the adaptor proteins McsB, MecA, and YpbH (Kirstein et al. 2009, Battesti and Gottesman 2013). McsB plays an important role during stress response as it is an adaptor protein and an arginine kinase that can target proteins, including CtsR, for degradation (Kirstein et al. 2007, Fuhrmann et al. 2009, Elsholz et al. 2017). In addition, unfolded proteins can also become phosphorylated by McsB, generating a degron, which can be directly recognized and processed by ClpCP (Trentini et al. 2016). Interestingly, the arginine phosphatase YwlE counteracts and controls the McsB kinase activity and cellular protein arginine phosphorylation state. This unusual stress-induced protein modification generated by the McsA-activated McsB kinase and controlled by the YwlE phosphatase plays important general and regulatory roles in the PQC of *B. subtilis* (Elsholz et al. 2017). ClpE is also induced under severe heat stress conditions by CtsR; however, no adaptor proteins have been identified so far (Derre et al. 1999a, Gerth et al. 2004, Elsholz et al. 2017). The maintenance of CtsR activity seems to be controlled by both ClpC and ClpE, as a *clpE* mutant shows delayed CtsR-dependent repression after heat shock (Miethke et al. 2006).

#### General stress response by sigma B

CtsR- and HrcA-dependent responses aim to repair misfolded or remove damaged and aggregated proteins. However, other proteins are also crucial for the heat shock response. The general stress SigB controls around 200 genes composing the general stress response (Haldenwang and Losick 1979, Nannapaneni et al. 2012). The activity of SigB is tightly regulated by the phosphorylation status of RsbV, which in turn is regulated by multiple reversible phosphorylation-dependent partner switching events (Fig. 1C) (Hecker et al. 2007). During exponential phase/nonstress conditions, SigB is sequestered by its antisigma factor RsbW. The anti-anti-sigma factor RsbV is phosphorylated under these conditions (RsbV∼P), unable to bind to RsbW. Upon stress, RsbV is dephosphorylated by one of the PP2C-type phosphatases RsbU or RsbP depending on the type of stress encountered. This dephosphorylation changes the binding affinity of RsbV to RsbW, releasing SigB from it. Free SigB binds to the core RNA polymerase and changes the promoter selectivity of the holoenzyme (Haldenwang 1995). Over time, the kinase RsbW phosphorylates RsbV, releasing it and reinstating SigB inactivation. SigB is activated as a response to multiple stresses by two different mechanisms (Voelker et al. 1995): During environmental stress (e.g. heat, acid, and ethanol), SigB is activated via a 25S multiprotein complex called the stressosome, consisting of RsbS, RsbT, and the paralogues RsbRA, RsbRB, RsbRC, RsbRD, and YtvA. Upon stress, the kinase activity of RsbT is stimulated, and RsbS and RsbRA are phosphorylated, allowing the release of RsbT from the complex to interact with RsbU (Kim et al. 2004, Delumeau et al. 2006, Gaidenko et al. 2006). This allows the dephosphorylation of RsbV∼P and the release of SigB from RsbW. It is known that the ribosomal protein L11 and GTPases of the ribosome assembly pathway, such as Obg, are involved in stress sensing for SigB by an unknown mechanism (Scott and Haldenwang 1999, Zhang et al. 2001). Energy stress, such as starvation for glucose or phosphate, can also activate the general stress response. In this case, the PP2C-type phosphatase RsbP forms a complex with RsbQ. RsbP additionally contains a PAS domain that can sense the cell’s energy potential. RsbPQ dephosphorylates RsbV∼P, indirectly activating SigB. A recent comparative transcriptome and proteome analysis of stringent and heat stress response in *B. subtilis* indicated a possible role of (p)ppGpp in SigB activation (Schäfer et al. 2020). Another mechanism for SigB activation is via cold shock, which is independent of RsbV, RsbP, and RsbU (Brigulla et al. 2003). Many genes in the SigB regulon are involved in stress response, and some of these genes, like *clpC* and *clpP*, are also regulated by other stress response mechanisms. For many of the genes in the SigB regulon, however, the exact function is still elusive, but it has been shown that they are important to cope with various stresses (Hecker et al. 1996, 2007).

#### The transcription factor Spx is important for heat shock response

The transcription factor Spx regulates thiol- and oxidative stress response, whose regulon consists of more than 200 genes (Rochat et al. 2012). However, it was demonstrated that Spx is also a major regulator of heat shock response, and *spx* mutants fail to develop thermotolerance (Runde et al. 2014, Schäfer et al. 2018).

Spx activity and stability are tightly regulated on a transcriptional and post-translational level (Fig. 1B). The transcription of *spx* is under the control of the transcriptional regulators PerR (oxidative stress), SigB, and SigM (cell wall stress), resulting in transient accumulation of Spx (Höper et al. 2005, Jervis et al. 2007, Leelakriangsak et al. 2007). Importantly, during nonstress conditions, the adaptor protein YjbH targets Spx for degradation by ClpXP. Upon heat or oxidative stress, YjbH relocates to subcellular aggregates, resulting in Spx stabilization and accumulation and enabling its activity as a transcription factor (Nakano et al. 2003b, Larsson et al. 2007, Garg et al. 2009). Another layer of post-translational regulation was observed with the identification of the antiadaptor protein YirB, whose expression is induced upon cell wall stress. YirB inhibits the YjbH-mediated targeting of Spx for degradation (Rojas-Tapias and Helmann 2018).

In the absence of ClpX, ClpCP, with its adaptor protein MecA, can degrade Spx. Additional mechanisms for regulation have been proposed involving arginine phosphorylation and dephosphorylation by McsB and YwlE (Rojas-Tapias and Helmann 2019, Schäfer and Turgay 2019). Spx can be activated via the generation of a disulfide bond by oxidation (Nakano et al. 2003a), which could also be relevant during heat shock response (Rojas-Tapias and Helmann 2019).

The transcriptional activation of genes by Spx is important for heat shock response and crucial for developing thermotolerance (Runde et al. 2014). Spx by interacting directly with the RNA polymerase activates expression of (redox) chaperones but at the same time inhibits the transcription of rRNA, ribosomal proteins (r-proteins), and competence and motility genes (Rochat et al. 2012, Molière et al. 2016, Schäfer et al. 2018). Since Spx binds to the C-terminal domain of the RNA polymerase alpha-subunit, it is also inhibiting the interaction of the alpha-subunit with other activators (Nakano et al. 2001, 2003b). Additionally, by enhancing the recognition of some promoters, Spx can activate the transcription of stress response genes. In a negative feedback loop, Spx positively regulates *clpX* and *yjbH* (Larsson et al. 2007).

It is important to emphasize that the ability of Spx not only to upregulate the redox chaperones but also to downregulate the expression of translation-involved genes is relevant for maintaining cellular protein homeostasis, to avoid the formation of aggregates and relieving some load on the PQC system during heat stress (Schäfer et al. 2018).

## (p)ppGpp as part of stress response in *B. subtilis*

The stringent response is the physiological response caused by the accumulation of the nucleotide second messengers (p)ppGpp. Increased concentrations of these alarmones were initially observed as a consequence of amino acid starvation. However, it is known that they are also synthesized as a response to multiple other stresses, e.g. heat shock, salt stress, cell wall stress, and nutrient limitations (Schäfer and Turgay 2019, Schäfer et al. 2020). Additionally, (p)ppGpp is involved in the development of persister cells, motility, competence, biofilm formation, and virulence for some pathogenic bacteria (Liu et al. 2015, Bange et al. 2021, Irving et al. 2021).

**Figure 2.**
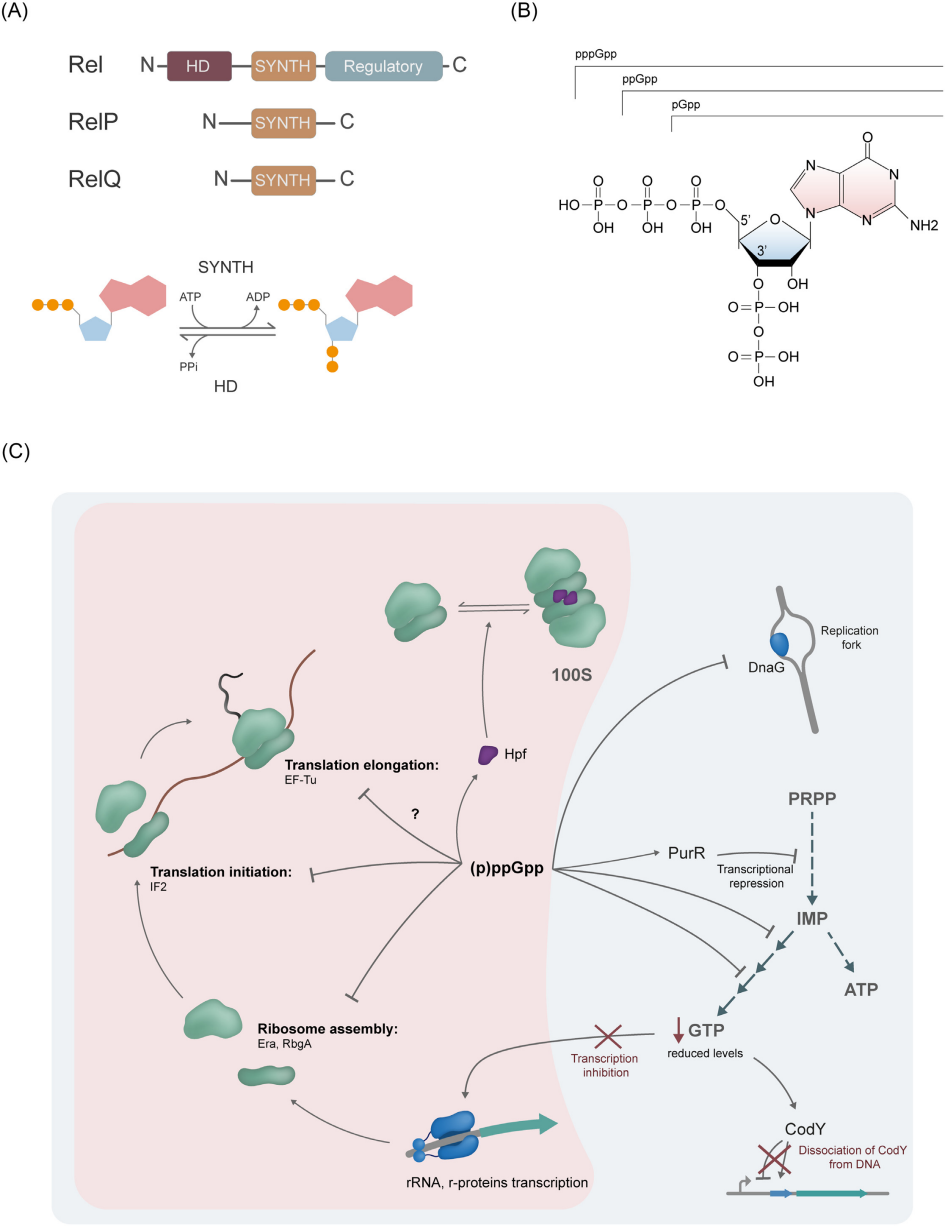
(p)ppGpp and its role in heat and stringent responses. **(A)** Domain organization of *B. subtilis* (p)ppGpp synthetases. HD: alarmone hydrolase domain, SYNTH: (p)ppGpp synthetase domain. Rel also contains regulatory domains in the C-terminal region. The SYNTH domain transfers a pyrophosphate group from ATP to a GTP, GDP, or GMP molecule to generate pppGpp, ppGpp, or pGpp, respectively. This group can be removed by the HD domain from Rel. **(B)** Chemical structure of (p)ppGpp alarmones. In purple is the guanine base; in blue is the ribose ring. The pyrophosphate transferred from ATP is attached to the 3′C of the ribose. **(C)** Targets of (p)ppGpp in *B. subtilis*. (1) The red background highlights possible (p)ppGpp interactions during heat shock response with GTPases involved in ribosome assembly, translation initiation, and elongation. These alarmones can also upregulate Hpf expression, which helps in ribosomal hibernation by forming 100S particles. (2) During stringent conditions, (p)ppGpp can also inhibit the primase DnaG, transcriptionally repress the IMP synthesis pathway via PurR; and directly inhibit enzymes that transform IMP to GTP. This inhibition results in decreased cellular GTP levels that reduce rRNA and r-proteins transcription and dissociation of CodY from its target promoters.

These alarmones are synthesized by members of the RelA/SpoT homolog (RSH) protein superfamily (Fig. 2A). Synthetases of this RSH family can transfer a pyrophosphate group from ATP to the 3′ carbon of either GTP, GDP, or GMP, generating the molecules pppGpp, ppGpp, and pGpp, respectively (Fig. 2B). *Bacillus subtilis* contains one long RSH protein, Rel, which can synthesize and hydrolyze (p)ppGpp. The synthetase and hydrolase domains are located in the N-terminal of Rel, and their activities are mutually exclusive (Hogg et al. 2004). Under nonstress conditions, Rel is located in the cytoplasm in a hydrolase ‘ON’ state. Rel binds to the A-site of the 70S ribosomes and, together with the deacylated tRNAs and adopts a synthetase ‘ON’ state; however, different models for the order of the events exist. A recent publication suggests that Rel first localizes at the ribosome, and its interaction is stabilized by the binding of the deacylated tRNA (Takada et al. 2021). Other models suggest that Rel first interacts with the deacylated tRNA and is then recruited to the ribosomes (Arenz et al. 2016, Brown et al. 2016, Loveland et al. 2016, Pausch et al. 2020). There is evidence that also during other stresses, such as heat and oxidative stress, Rel is recruited by a similar mechanism to the ribosome (Schäfer et al. 2020).

It should be noted that Rel synthetase/hydrolase activity can also be influenced by interaction with regulatory proteins from other pathways in various bacteria (Boutte and Crosson 2013, Hallez et al. 2017). Such interaction has been suggested in *B. subtilis* for competence development via interaction with ComGA (Hahn et al. 2015). Recent papers have shown that the activity of Rel can be modulated by the c-di-AMP binding protein DarB. In the absence of c-di-AMP, DarB can interact with Rel and stimulate the synthesis of (p)ppGpp. These findings link the stringent response to c-di-AMP, another second messenger (Krüger et al. 2021, Heidemann et al. 2022, Ainelo et al. 2023).

Additionally, *B. subtilis* encodes two small alarmone synthetases (Sas) called RelP/SasA and RelQ/SasB that contain only a synthetase domain. These small synthetases were originally discovered because a *rel* mutant gains suppressor mutations in *relP* and *relQ* due to raised (p)ppGpp levels in the absence of the Rel hydrolase domain (Nanamiya et al. 2008, Srivatsan et al. 2008, Steinchen et al. 2015). The exact function of these small synthetases has not been elucidated in detail; however, the long RSH Rel seems to be the major (p)ppGpp synthetase activated during protein folding stress conditions (Schäfer et al. 2020). *In vitro* studies have demonstrated that RelQ is allosterically regulated by pppGpp (Steinchen et al. 2015). Additionally, it has been shown that *in vivo* RelQ protein levels can be detected throughout the growth curve with a peak when cells are transitioning from exponential into stationary phase (Tagami et al. 2012). RelP is transcriptionally upregulated in response to cell wall stress, which suggests a possible role in stress response (Eiamphungporn and Helmann 2008, D’Elia et al. 2009, Libby et al. 2019).

### (p)ppGpp can directly downregulate translation at multiple steps

During stringent conditions, Rel synthesizes (p)ppGpp at the ribosome, leading to a local increase of (p)ppGpp levels. (p)ppGpp can bind to multiple GTPases involved in ribosome assembly, such as RbgA (Corrigan et al. 2016) or translation initiation, such as IF2 (Diez et al. 2020). Inhibition of translation elongation by binding to EF-Tu was observed for *E. coli* (Rojas et al. 1984). Due to the structural similarity, EF-Tu from *B. subtilis* might also bind (p)ppGpp. By binding to the translation GTPases, the alarmone can inhibit translation at multiple steps and, therefore, can immediately slow down the translation rate.

Additionally, (p)ppGpp is also involved in the formation of hibernating 100S ribosomes, which protects nontranslating ribosomes. Here, the expression of the hibernation factor Hpf, which enables the disome formation, is induced in the presence of (p)ppGpp, most likely via CodY (Tagami et al. 2012, Belitsky and Sonenshein 2013, Beckert et al. 2017, Schäfer et al. 2020). It was also demonstrated that (p)ppGpp could bind to the SRP GTPase Ffh, thereby inhibiting the SRP receptor targeting complex formation and inhibiting membrane insertion and protein secretion via SRP (Czech et al. 2022).

### (p)ppGpp can directly inhibit enzymes of the purine biosynthesis on the protein as well as the transcriptional level

During an amino acid downshift, it is crucial for the survival of the cells to quickly synthesize amino acids to facilitate protein biosynthesis. Phosphoribosyl diphosphate (PRPP) is required for the synthesis of multiple amino acids as well as nucleotides. Similar to the translation inhibition, (p)ppGpp can inhibit the enzymes facilitating GTP biosynthesis at multiple steps in the *de novo* pathway (GuaB, Gmk) and the salvage pathways (HprT, Xpt) (Lopez et al. 1981, Kriel et al. 2012, Anderson et al. 2019, 2020). Additionally, it was recently shown that in *B. subtilis*, (p)ppGpp can bind to the transcriptional repressor PurR that regulates the expression of purine biosynthesis genes. PRPP inhibits PurR binding to target promoter regions, acting as an inducer (Weng et al. 1995). (p)ppGpp acts as an anti-inducer by competing with PRPP for binding PurR, thereby inhibiting the PRPP-induced de-repression of the PurR regulon (Anderson et al. 2021). This is the first example of direct regulation of a transcription factor by (p)ppGpp in Firmicutes.

It is worth noting that another ancient and highly conserved stress-induced second messenger, diadenosine tetraphosphate (Ap4A), interacts in *B. subtilis* with the inosine-5′-monophosphate dehydrogenase (IMPDH, GuaB), a key branching point enzyme for the biosynthesis of adenosine or guanosine nucleotides, and can thereby regulate GTP synthesis. This could represent an interesting coregulation of two stress-induced alarmone second messengers on nucleotide synthesis in *B. subtilis* (Giammarinaro et al. 2022).

### Reduction in GTP levels inhibits transcription via CodY and RNA polymerase

In *E. coli*, (p)ppGpp can directly interact with RNA polymerase at the interface of ω and β’ subunits and in the interface between the transcription factor DksA and β’ subunit. The ppGpp interacting with DksA and the RNA polymerase initiates by an unknown mechanism the transcriptional program of stringent response in *E. coli* (Ross et al. 2013, 2016, Travis and Schumacher 2022). A comparable mechanism was not observed in *B. subtilis*, so far. However, it was observed that the transcription of promoters that initiate with guanosine is sensitive to intracellular GTP concentration and is strongly reduced upon GTP depletion. Transcription of some rRNA and ribosomal proteins (r-proteins) is inhibited by this mechanism indirectly in response to high (p)ppGpp levels (Krásný and Gourse 2004, Krásný et al. 2008).

Additionally, the depletion of GTP levels influences the activity of the transcription factor CodY, which regulates more than 200 genes (Belitsky and Sonenshein 2013, Brinsmade et al. 2014). CodY requires branched-chain amino acids and GTP to bind and repress or activate its regulon (Ratnayake-Lecamwasam et al. 2001, Shivers and Sonenshein 2004). During stringent conditions, the GTP levels decrease through (p)ppGpp, reducing CodY binding and derepressing its regulon. CodY regulates many genes for amino acid biosynthesis and plays a crucial part in stringent response during amino acid starvation and entry into the stationary phase (Geiger and Wolz 2014).

### Role of (p)ppGpp during proteotoxic stress—it is all about the aggregates

**Figure 3.**
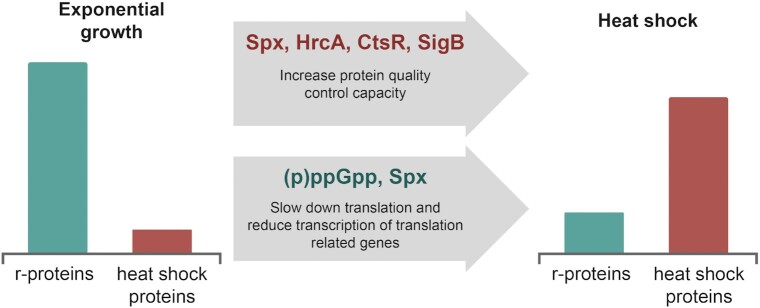
Transcriptional and translational adaptation of *B. subtilis* to heat shock. Upon a sudden increase in temperature, the transcription factors Spx, HrcA, CtsR, and SigB induce the transcription of AAA+ proteases, (redox)chaperones, and other heat shock proteins to aid against protein misfolding and aggregation. At the same time, Spx downregulates r-protein biosynthesis and (p)ppGpp inhibits translation GTPases, slowing down protein synthesis, and thereby reducing the load to the cellular PQC system.

It was recently demonstrated that (p)ppGpp levels exhibit a short peak upon exposure to a strong heat shock *B. subtilis*, returning to basal levels after 15 minutes (Schäfer et al. 2020). Interestingly, (p)ppGpp deficient cells [(p)ppGpp^0^] are more sensitive to heat shock. Additionally, a (p)ppGpp^0^ strain shows more aggregate formation and a higher translation rate during heat shock than the wildtype. Furthermore, the deletion of *rel*, which exhibits high (p)ppGpp levels because these cells cannot hydrolyze (p)ppGpp, has a lower translation rate. Interestingly, proteomic analyses reveal that chaperone levels are still upregulated even though the translation is downregulated in this strain (Fig. 3). This could indicate a selective mechanism for translation with high (p)ppGpp levels (Schäfer et al. 2020). Indeed, an *in vitro* study suggests modulation of the proteome in the presence of (p)ppGpp depending on the mRNA structure (Vinogradova et al. 2020).

Additionally, transcriptomic and proteomic analyses reveal that multiple ribosomal proteins were downregulated in a (p)ppGpp-dependent manner upon heat shock, and RT-qPCR also revealed a partially (p)ppGpp-dependent downregulation of rRNA during heat shock. Interestingly, the GTP levels were not directly affected upon the short peak of (p)ppGpp, and the CodY regulon was still repressed. Therefore, one can conclude that during heat shock response, (p)ppGpp inhibits translation directly and has a mild effect on the transcription of rRNA and r-protein genes, preventing the formation of toxic aggregates. However, Spx also represses rRNA transcription upon heat stress (Schäfer et al. 2018). A (p)ppGpp^0^ ∆*spx* strain exhibits a strong growth defect, which is not present in the single mutants when grown at 50°C (Schäfer et al. 2020). This indicates a functional interplay between the two stress responses, and while defects in one system can be dispensable, defects in both systems result in severe stress sensitivity. Even in (p)ppGpp^0^ ∆*spx* mutant cells, downregulation of rRNA and r-proteins happens to some extent during heat shock, indicating that there might be additional mechanisms to control their transcription. The Spx regulon has overlaps with the SigB or the CtsR regulon that could serve as another safety net. Furthermore, (p)ppGpp levels seem connected to the induction of general stress response, as the activation of some SigB-activated genes during heat shock response is reduced in a (p)ppGpp^0^ strain (Schäfer et al. 2020).

The accumulation of (p)ppGpp is a swift response, and inhibition of protein biosynthesis clearly prevents aggregate formation. When exposed to a strong heat shock, misfolding and unfolding of proteins likely happens faster than the transcription of heat shock proteins. In this case, a short peak (p)ppGpp can act as an emergency brake to slow down translation and reduce the load on the PQC until the chaperone and protease systems become fully available.

### Open questions

(p)ppGpp is a versatile regulator of the stress response. Depending on its concentration and duration, the strength of the response differs. The stringent response has been studied for many years since the discovery of these alarmones. Nevertheless, many questions are still not answered. It is not known whether or how Rel senses protein folding stress. A possible scenario could be that stresses like heat could interfere with tRNA activity or charging, which could increase deacylated tRNAs inducing the stringent response. The translation is slowed down during heat stress, but how is the expression of chaperones then still upregulated? Interestingly, during heat shock, a downregulation of some rRNA and r-proteins could be observed even though the GTP levels are not affected (Schäfer et al. 2020). This could indicate that other transcriptional regulators could also be affected directly by (p)ppGpp, such as the recently discovered PurR regulation (Anderson et al. 2021).

Future research will be very important to fully understand the intricate involvement and interconnection of second messenger activity, and the control of their synthesis and degradation, which allows the cell to orchestrate a fast stress response on many different cellular and regulatory levels. Apparently these pathways can be connected to various distinct stress or regulatory pathways allowing the adaptation of bacterial organisms to different niches in fast changing environments, which is also necessary for specific virulence mechanisms of pathogenic bacteria.
